# In Ovo Delivered Toll-Like Receptor 7 Ligand, Resiquimod Enhances Host Responses against Infectious Bronchitis Corona Virus (IBV) Infection

**DOI:** 10.3390/vaccines8020186

**Published:** 2020-04-15

**Authors:** Upasama De Silva Senapathi, Mohamed Aboelkhair, Kekungu Puro, Mariam Ali, Aruna Amarasinghe, M. Sarjoon Abdul-Cader, Guido Van Marle, Markus Czub, Mohamed Faizal Abdul-Careem

**Affiliations:** 1 Department of Ecosystem and Public Health, Faculty of Veterinary Medicine, University of Calgary, Health Research Innovation Center 2C53, 3330 Hospital Drive NW, Calgary, AB T2N 4N1, Canada; yaseshwari.desilvase@ucalgary.ca (U.D.S.S.); mariam.ali3467@gmail.com (M.A.); arunavetlk@gmail.com (A.A.); mohamedsarjoon.moham@ucalgary.ca (M.S.A.-C.); mmczub@ucalgary.ca (M.C.); 2 Virology Department, Faculty of Veterinary Medicine, University of Sadat City, 999060 Sadat, Egypt; maboelkhair2004@yahoo.com; 3 Division of Animal Health, ICAR Research Complex for NEH Region, Umiam, 793103 Meghalay, India; akulepuro@rediffmail.com; 4 Department of Microbiology, Immunology and Infectious Diseases, Cumming School of Medicine, University of Calgary, 3330 Hospital Drive NW, Calgary, AB T2N 4N1, Canada; vanmarle@ucalgary.ca

**Keywords:** in ovo, resiquimod, avian macrophage, CD8α+ cell, infectious bronchitis virus, interleukin 1β, interferon γ

## Abstract

Toll-like receptor (TLR) 7 ligand, resiquimod, has been studied as an adjuvant and antiviral agent against several pathogens in chicken. Yet, the effectiveness of resiquimod against infectious bronchitis virus (IBV) infection has not been evaluated. In this study, we investigated the effectiveness of resiquimod delivered pre-hatch (in ovo) against IBV infection post-hatch identifying key mechanisms involved in resiquimod driven immune activation. First, we found an upregulation of interleukin (IL)-1β and interferon (IFN)-γ mRNA levels and considerable expansions of macrophage and cluster of differentiation (CD) 8α+ T cell populations in lungs of chicken as early as day one post-hatch, following pre-hatch delivery of resiquimod. Second, we observed that resiquimod was able to act as an adjuvant when resiquimod was delivered pre-hatch along with an inactivated IBV vaccine. Finally, when the resiquimod pretreated one-day-old chickens were infected with IBV, reduction in viral shedding via oral and fecal routes was observed at 3 days post- infection. Overall, this study shows that the pre-hatch delivered resiquimod increases cell-mediated immune responses in lungs with an advantage of reduction in IBV shedding.

## 1. Background

Infectious bronchitis (IB) is one of the major respiratory diseases that impacts chickens (*Gallus gallus domesticus*) worldwide [1,2,3]. In spite of immunization, IB remains a considerable cause of economical loss to the poultry industry [4]. Therefore, alternative strategies that aim to boost the host responses against IBV are increasingly being investigated.

Toll-like receptor (TLR) ligands, a group of innate immune response modifiers have become worthy candidates that have been exploited to enhance innate immune responses in hosts against a number of pathogens [5]. TLRs are a subset of germline-encoded pattern recognition receptors found in mammals, birds, and fish. Upon infection, TLRs are able to recognize and bind to pathogen-associated molecular patterns (PAMPs), which are highly conserved molecular structures present in microbes. These PAMP–TLR receptor interactions trigger a cascade of intracellular interactions that results in the upregulation of a range of genes responsible for innate immune activation. Thirteen different types of TLRs have been identified in mammalian cells. Of those, TLR3, TLR4, TLR5, and TLR7, which are found in birds, are comparable to mammalian counterparts and TLR1La, TLR1Lb, TLR2a, TLR2b, TLR15 appear to be present only in birds [5,6], whereas TLR21 is present in both birds and fish [7].

Among the TLRs, TLR3, 7 and 21 in mammals and birds are known to be localized within the endosomal compartment [5]. TLR7 exclusively recognizes and binds with either ssRNA components that are rich in GU (guanosine-uridine) or poly-U rich sequences or their synthetic analogs, resiquimod, and imiquimod [8]. Resiquimod, one of the above mentioned imidazoquinoline compounds is a potent synthetic TLR7 and TLR8 agonists that stimulate the production of type I andⅡinterferons (IFNs), interleukin (IL)-4, IL-12, IL-1β, inducible nitric oxide synthase (iNOS), and tumor necrosis factor alpha (TNF-α) [9,10,11,12]. 

The ability of these synthetic analogs to be delivered in ovo (embryo day 18 (ED18)), as a standalone agent or as a vaccine adjuvant, to provide prophylaxis has several advantages over conventional delivery methods [13,14]. These advantages along with the superior ability of resiquimod rather than imiquimod to induce innate immune cytokines [8] have prompted investigators to direct studies in probing the potential of resiquimod against avian viruses such as infectious bursal disease virus (IBDV) [10], infectious laryngotracheitis virus (ILTV) [15], and avian influenza virus (AIV) [16,17]. Parenterally administered resiquimod is also known to act as an adjuvant against IB and Newcastle disease (ND) viral vaccines [12,18]. However, use of resiquimod as a prophylactic standalone agent for a post-hatch IBV challenge in chickens or adjuvant effect with inactivated vaccine when delivered in ovo has not been investigated previously. Therefore, we aimed at delivering resiquimod pre-hatch (in ovo) at ED18 to investigate the immune enhancement, particularly cell-recruitment and cell-mediated immune response in lungs and to ascertain the level of reduction in IBV genome load provided subsequent to post-hatch IBV infection.

## 2. Materials and Methods 

### 2.1. Animals

The use of specific pathogen free (SPF) eggs, embryos, and live chickens in all our experiments have been approved by the Health Science Animal Care Committee (HSACC) (AC13-0291). The SPF eggs were purchased from the Canadian Food Inspection Agency (CFIA), Ottawa and incubated in digital egg incubators (Kingsuromax 20 and Rcom MARU Deluxe max, Autoelex Co., Ltd., GimHae, GyeongNam, Korea) according to the manufacturer’s instructions. The chickens used in experiments were housed in high containment poultry isolators at the prion/virology animal facility, Health Research Innovation Center (HRIC), University of Calgary with ad libitum access to food, water, and necessary veterinary care.

### 2.2. Virus and TLR Ligand

IBV Massachusetts (M) 41 strain purchased from American Type Culture Collection (ATCC, Manassas, Virginia, United States) was used in this study. End point dilution assay was employed to assess the viral titers using embryo day (ED)9 SPF eggs and expressed as 50% embryo infectious dose (EID_50_) [19]. The synthetic analog of ssRNA, resiquimod, TLR7 ligand was purchased from Selleckchem (Houston, TX, USA).

### 2.3. Experimental Design

#### 2.3.1. Evaluation of Cellular and Cytokine Responses in Lungs Following In Ovo Resiquimod Treatment

To determine the mechanisms of action of in ovo delivered resiquimod in young chickens, ED18 SPF eggs were inoculated with either resiquimod (400 µg in 200 µL phosphate buffered saline or PBS (200 µL PBS alone) and incubated until hatch. On the day of hatch, coinciding with the time of potential pathogen encounter, chickens from both groups (resiquimod pretreated = 6, PBS pretreated = 6) were euthanized and lung tissue preserved in Optimum Cutting Medium (OCT, Leica Biosystems, Wetzlar, Germany) for immunofluorescent assay, in 10% neutral buffered formalin for histopathological examination, and in RNASave^®^ (Biological Industries, Beit Haemek, Israel) for cytokine mRNA analysis.

#### 2.3.2. Evaluation of Adjuvant Effect of Resiquimod

To determine the adjuvant effect of resiquimod in young chickens, ED18 SPF eggs were inoculated with either resiquimod (ssRNA) plus inactivated IBV vaccine (100 µg of resiquimod + 5 µg of inactivated IBV vaccine in 200 µL PBS), inactivated IBV vaccine (5 µg in 200 µL PBS), allantoic fluid (AF) (200 µL/egg) as vaccine control, and PBS (200 µL/egg) as experimental control and incubated until hatch. In ovo delivery was carried out as described previously [20]. On the day of hatch, the chickens (n = 3/group) were transferred to high containment animal isolators, reared with ad libitum feed and water. On day 12 post-hatch (12 dph), the chickens were humanely euthanized, and the lungs and spleens were collected. Subsequently, the mononuclear cells were isolated as described previously [21,22] for in vitro culture. The cells were seeded in 96-well plates with each sample (n = 3/group) in triplicate. Cells derived from lung tissue and the spleen were plated separately for the in vitro experiment. A subset of cells for both the lung and spleen cell plates were stimulated with 5 µg of inactivated IBV vaccine and another subset with PBS as the control. The cells were stimulated for 48 h at 40 °C with 5% CO_2_. The supernatant was collected, and interferon (IFN)-γ concentration was estimated by enzyme linked immunosorbent assay (ELISA) using chicken IFN-γ cytoset kit #CAC 1233 (Life Technologies Corporation, Frederick, MD, USA) following the manufacturer’s instruction. 

#### 2.3.3. Evaluation of IBV Genome Loads Following IBV Infection of Resiquimod Pretreated Chickens

In ovo TLR ligand delivery was carried out as described previously [20]. Four hundred µg of resiquimod diluted in 200 µL of PBS was inoculated per SPF egg on ED18 via allantoic sac route (n = 13), whereas the control received 200 µL of PBS alone (n = 15). On day 1 post-hatch, the chickens (n = 10) from the resiquimod pretreated group and PBS pretreated group (n = 12) were infected with IBV M41 strain intratrachealy at a dose rate of 2.75 × 10^4^ EID_50_ per bird [23,24] and the remaining birds were maintained as uninfected controls (Resiquimod control = 3, PBS control = 3). At 3 and 7 dpi, oropharyngeal and cloacal swabs were collected from infected birds using Puritan^®^ Unitranz-RT transport system (Puritan Medical Products LLC Guilford, Maine, USA) and stored in −80 °C until analyzed.

### 2.4. Techniques

#### 2.4.1. RNA Extraction and Complementary DNA (cDNA) Synthesis

RNA extraction of oropharyngeal and cloacal swabs was performed using E.Z.N.A.^®^ viral RNA kit (Omega Bio-tek Inc., Norcross, GA, USA) as per the manufacturer’s guidelines. Total RNA from the lungs was extracted using Trizol reagent (Invitrogen, Canada Inc., Burlington, ON, Canada) following the manufacturer’s protocol. Extracted RNA concentration was measured using a Nanodrop1000 spectrophotometer (ThermoScientific, Wilmington, DE, USA) at 260 and 280 nm wavelength absorbance. High Capacity cDNA Reverse Transcription Kit (Invitrogen Life Technologies, Carlsbad, CA, USA) was used as per the manufacturer’s guidelines to reverse transcribe 2 µg of total RNA from tissue samples and 200 ng of total RNA from swab samples.

#### 2.4.2. Real-Time Reverse Transcriptase Polymerase Chain Reaction (RT-PCR) Assay

The cytokine mRNA expression and IBV genome load quantification was carried out as described previously [24]. In brief, IBV nuclear (N) gene and cytokine genes were quantified using Fast SYBR^®^ Green Master Mix (Invitrogen, Burlington, ON, Canada) in a RT-PCR assay. The target genes were amplified using gene specific primers (Supplementary Table S1). The Thermal Cycler (CFX96-C1000, Bio-Rad Laboratories, Mississauga, ON, Canada) conditions were 95 °C for 20 s (s) of pre-incubation, 95 °C for 3 s, and 60 °C for 30 s for 40 amplification cycles. A melting curve analysis was performed between 95 °C and 65 °C, with a 0.5 °C raise in temperature every 5 s. Fluorescent signal acquisition was performed at 60 °C for 30 s.

#### 2.4.3. Immunofluorescent Assay

Immunofluorescent Assay (IFA) to detect macrophages (KUL01+), CD4+ T cells, and CD8α+ T cells was performed as described previously [24]. In brief, the OCT preserved lung tissue sections were fixed in cold acetone for 5 min (min) and blocked in 5% goat serum diluted in a Trisma buffered saline (TBS) buffer at room temperature for 30 min. The primary antibodies, the mouse monoclonal antibody specific for chicken macrophages/monocytes, KUL01+ (Southern Biotech, Birmingham, AL, USA), CD8α (CT-8, Southern Biotech, Birmingham, AL, USA), were used in a 1:200 dilution in a 5% goat serum for 30 min. Then, the secondary antibody, goat anti-mouse IgG (H + L) conjugated with Dylight^®^ 550 (Red fluorescence, Bethyl Laboratories Inc., Montgomery, TX, USA) was used for 1 h. For CD4+ staining, chicken CD4 antibody (CT-4, Southern Biotech, Birmingham, AL, USA) was used as the primary antibody followed by biotinylated goat anti-mouse IgG (H + L) (Southern Biotech, Birmingham, AL, USA) used in a 1:250 dilution. Dylight^®^ 488 (green fluorescence) streptavidin was used as a final step followed by mounting the slides with Vectashield^®^ mounting medium with DAPI (Vector Laboratories Inc., Burlingham, CA, USA).

#### 2.4.4. Inactivation of IBV and Determination of Protein Concentration

The stock IBV virus propagated in ED9 SPF eggs was concentrated by ultra-centrifugation at 55,000× *g* for 3 h at 4 °C. Then, one-tenth of the volume (after resuspending the pellet) was inactivated using formalin with a final concentration of 0.1% [25]. The protein concentration of the vaccine was determined using Bio-Rad Dc Protein Assay kit (cat# A 500-0113, B 500-0114, C 500-0115, Bio-Rad Laboratories, Life sciences group, Hercules, CA, USA) with bovine serum albumin (BSA) as a standard.

#### 2.4.5. Mononuclear Cell Isolation from Lung and Spleen

The collected spleen and lungs were rinsed multiple times in cold Hank’s balanced salt solution (HBSS) to get rid of blood contamination. The spleens were homogenized, filtered through a 40 mm cell strainer (VWR, Edmonton, AB, Canada) and the cells were collected. The lungs were minced using sterile scalpel and forceps, to approximately 5 mm fragments and soaked in 400 U/mL collagenase type I solution (Sigma-Aldrich, Oakville, ON, Canada) for 30 min at 37 °C. Dispersed cells and tissue fragments were separated using a 40 mm cell strainer. The cells were pelleted at 400 × g for 10 min (4 °C), followed by resuspension in HBSS and carefully layered onto Ficoll Paque PLUS (GE Health Care, Mississauga, ON, Canada) making sure not to disturb the Ficoll layer in a 15 mL conical tube at room temperature in 1:1 ratio. The layered cells were spun for 40 min at 400× *g* at 20 °C. The mononuclear cells were collected from the interface and pelleted, washed with HBSS, and the cells were suspended in complete Roswell Park Memorial Institute (RPMI) medium-1640 (RPMI-1640 supplemented with 10% heat inactivated fetal bovine serum, 100 U/mL penicillin and 100 µg/mL streptomycin), and the cells were counted. The lung and spleen mononuclear cells (1 × 10^6^ cells/well) in 120 uL of complete RPMI-1640 medium were seeded into 96-well plates (Greiner Bio-one GmbH-Frickenhausan, Germany).

### 2.5. Data Analyses

For the quantification of macrophages (KUL01+), CD4+ T cells, and CD8α+ T cells in lung tissue, 5 areas with the most positive fluorescent signals for KUL01+, CD4+ T cells, and CD8α+ T cells per tissue section were captured at 20× magnification along with corresponding nuclear stained (4′,6-diamidine-2′-phenylindole dihydrochloride, DAPI) fluorescent areas. The images were quantified using Image J software (National Institute of Health, Bethesda, MD, USA). Fluorescent intensities for Dylight^®^ 550 (KUL01+ and CD8α+ cells) and DyLight^®^ 488 (CD4+ cells) positive signals were expressed relative to total area (as estimated by nuclear fluorescent signal with DAPI) and given as a percentage.

### 2.6. Statistical Analyses

Group differences in viral loads in oropharyngeal and cloacal swabs were identified using linear mixed-effects model followed by a pairwise comparison using Tukey’s test in R statistical software (R studio version 1.0.153, Boston, MA, USA). For identifying differences in IFN-γ concentrations, the one-way analysis of variance (ANOVA) followed by Tukey’s multiple comparisons was used. A one-tailed t-test was used to identify differences among two groups. Data in graphs are shown in the original scale of measurements. However, due to non-normality and inability to satisfy model assumptions of some datasets, log transformation was applied to these prior to analysis. GraphPad Prism Software 5 (La Jolla, CA, USA) and R statistical software (R studio version 1.0.153, Boston, MA, USA) was used to perform model statistics.

## 3. Results

### 3.1. Immune Cell Recruitment Following in Ovo Delivery of Resiquimod 

**Figure 1:**
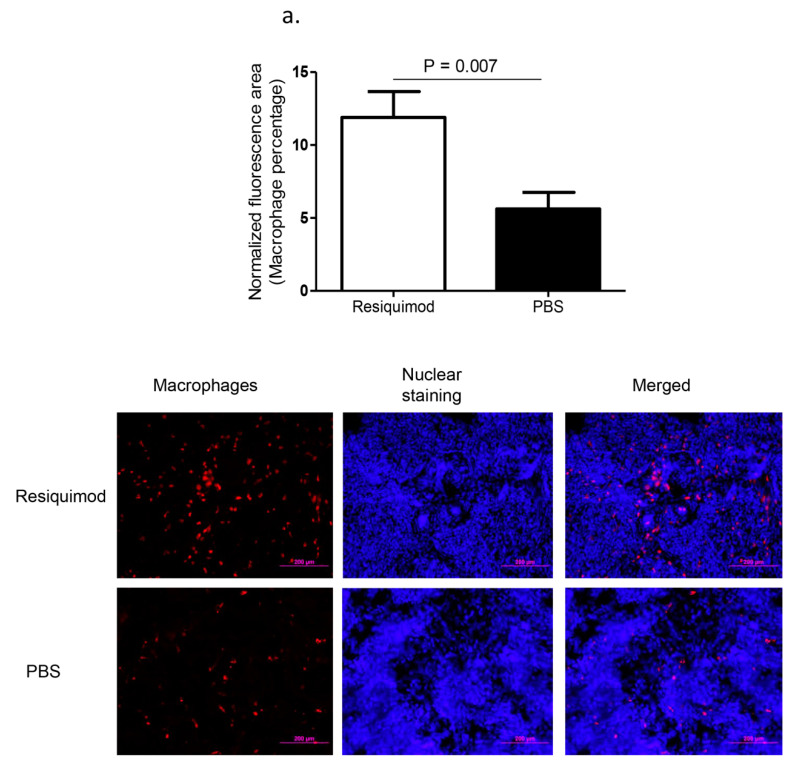

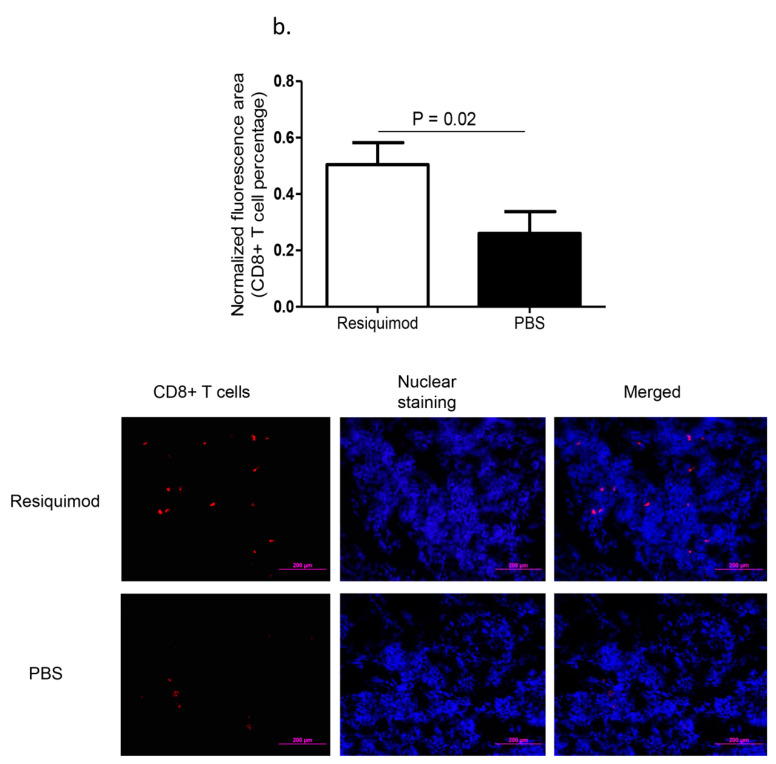

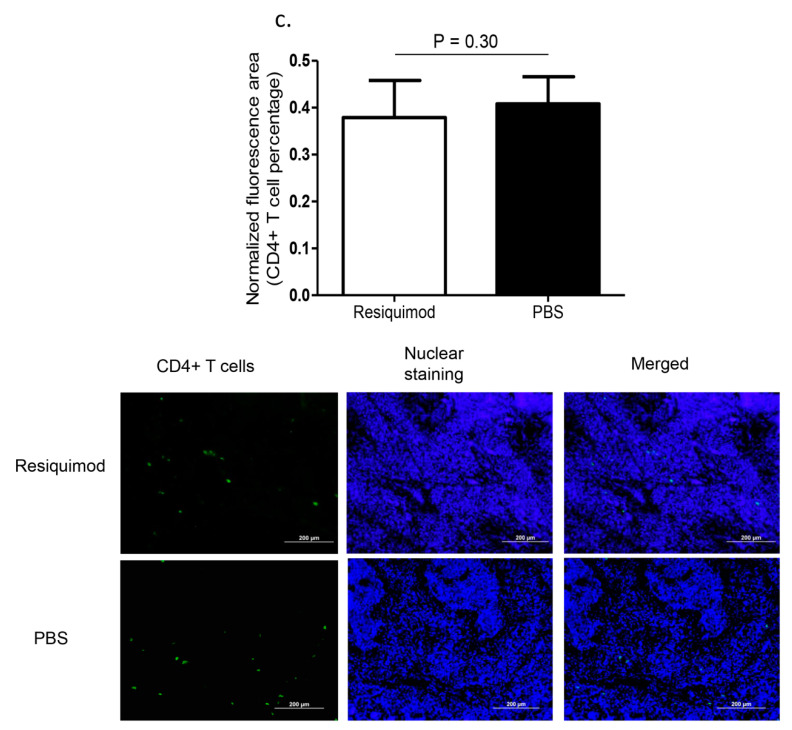
Immune cell recruitment following in ovo delivery of resiquimod or PBS. Embryo day 18 (ED18) specific pathogen free (SPF) eggs were treated with resiquimod (n = 6, 400 µg of resiquimod in 200 µL of PBS per egg) or PBS (n = 6, 200 µL of PBS per egg) and incubated to hatch. On the day of hatch, the birds were euthanized and lung tissue collected in Optimum Cutting Medium (OCT) compound for immunofluorescent assays. Quantitative data and representative images following immunofluorescent assay for lung (**a**) macrophages (red), (**b**) CD8α+ T cells (red), and (**c**) CD4+ T cells (green) are depicted. One-tailed Student’s t-test was performed to identify group differences. The differences were considered significant at *p* ≤ 0.05. The error bars represent ± standard error of mean (SEM).

Immunofluorescent assay performed on lung tissue obtained at day one post-hatch, revealed a significant increase in macrophage (*p* = 0.007, Figure 1a) and CD8α+ T (*p* = 0.02, Figure 1b) numbers in resiquimod pretreated chickens as compared with the PBS pretreated controls. However, CD4+ T cell numbers were not significantly different between resiquimod and PBS pretreated lungs (*p* > 0.05, Figure 1c).

### 3.2. IFN-γ, IL-1β, and iNOS mRNS Expression Levels Following In Ovo Delivery of Resiquimod

**Figure 2:**
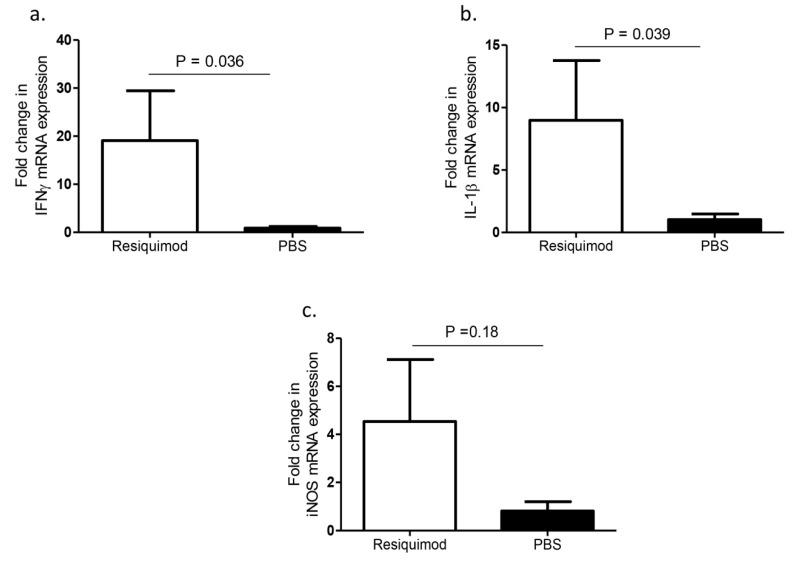
IFN-γ, IL-1β, and iNOS mRNA expression levels following in ovo delivery of resiquimod or PBS. ED18 SPF eggs were treated with resiquimod (n = 6, 400 µg of resiquimod in 200 µL of PBS per egg) or PBS (n = 6, 200 µL of PBS per egg) and incubated to hatch. On the day of hatch, birds were euthanized, and lung tissues collected to assess mRNA expressions of (**a**) IFN-γ, (**b**) IL-1β, and (**c**) iNOS. One-tailed Student’s t-test was performed to identify group differences. The differences were considered significant at *p* ≤ 0.05. The error bars represent ± SEM.

The mRNA expression levels of several cytokines in lung tissues of one-day-old chickens after pretreating ED18 SPF eggs with either resiquimod or PBS were compared. We found a significant upregulation of IFN-γ and IL-1β expression levels in the day-old lung tissue of resiquimod pretreated chickens as compared with controls (*p* = 0.036, *p* = 0.039, Figure 2a,b) and an increased trend in iNOS mRNA levels, although not statistically significant (*p* > 0.05, Figure 2c).

### 3.3. IBV Shedding via Oropharyngeal and Cloacal Routes Following Infection with IBV M41 in In Ovo Resiquimod Pretreated Chickens 

**Figure 3:**
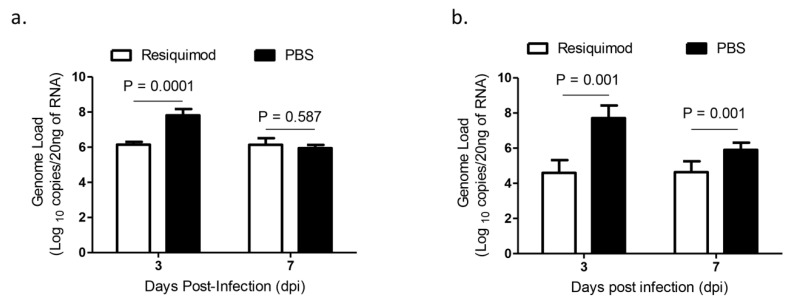
Infectious bronchitis virus (IBV) shedding via oropharyngeal and cloacal routes following infection with IBV M41 in in ovo resiquimod pretreated chickens. ED18 SPF eggs were treated with resiquimod (n = 10, 400 µg of resiquimod in 200 µL of PBS per egg) or PBS (n = 15, 200 µL of PBS per egg) and incubated to hatch. On the day of hatch, the chickens were infected with IBV M41 intratracheally. On 3 and 7 dpi (**a**) oropharyngeal and (**b**) cloacal swabs were used for the extraction of RNA and real-time PCR assay was used for the IBV genome quantification. Linear mixed-effects model followed by a pairwise comparison using Tukey’s test was used to identify group differences in oropharyngeal and cloacal IBV genome loads. The differences were considered significant at *p* ≤ 0.001. The error bars represent ± SEM.

The average IBV viral RNA loads of oropharyngeal and cloacal swabs were lower in resiquimod pretreated chickens at 3 dpi and in cloacal swabs at 7 dpi as compared with that of PBS pretreated chickens (*p* < 0.001, Figure 3a,b). However, we did not observe a significant difference in oropharyngeal IBV genome loads between the two groups at 7 dpi (*p* > 0.05, Figure 3a,b).

### 3.4. Adjuvant Effect of Resiquimod Following In Ovo Delivery

**Figure 4:**
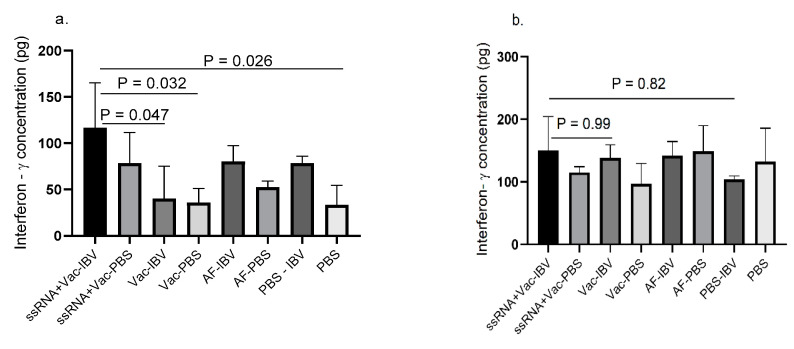
IFN-γ production in mononuclear cells of chickens that received in ovo resiquimod plus IBV killed vaccine. ED18 SPF eggs (n = 6 in each group) were treated with resiquimod + inactivated IBV vaccine (ssRNA + Vac) (100 µg of resiquimod + 5 µg inactivated IBV vaccine in 200 µL of PBS per egg), inactivated IBV vaccine (Vac) (5 µg inactivated IBV vaccine in 200 µL of PBS per egg), allantoic fluid (AF) (200 µL per egg), or PBS (200 µL per egg) and incubated to hatch. At 12 days post- immunization, the chickens (n = 3 per group) were euthanized and mononuclear cells harvested from (**a**) lung and (**b**) spleen, a subset of each group was stimulated with inactivated IBV vaccine (5 µg) and PBS control for 48 h. The IFN-γ production was measured by ELISA. The one-way ANOVA followed by Tukey’s multiple comparisons test was used to identify the statistical differences between various groups. The differences were considered significant at *p* ≤ 0.05 the bars represent mean± SEM.

The resiquimod (ssRNA) plus IBV killed vaccine group showed higher IFN-γ concentration which significantly differ (*p* < 0.05) from IBV killed vaccine alone and control groups in lung (Figure 4a). However, a statistically significant difference between the resiquimod (ssRNA) plus IBV killed vaccine group and the two control groups was not seen although a trend of lower IFN-γ production was seen as compared with the resiquimod (ssRNA) plus IBV killed vaccine group. No significant differences were observed between various groups in spleen (Figure 4b) although the concentrations were found to be higher than in lung.

## 4. Discussion

The study described here aimed at investigating the efficacy and involved mediators following pre-hatch delivery of resiquimod against IBV infection encountered post-hatch. First, we found that in ovo delivered resiquimod was effective in enhancing host response characterized by increased recruitment of macrophages and CD8α+ T cells and increased mRNA expression of IFN-γ and IL-1β in the lungs. Second, we found that the in ovo delivered resiquimod along with IBV killed vaccine were effective in enhancing IFN-γ production following restimulation with inactivated IBV. Finally, the enhanced host responses associated with reduction in IBV shedding early during the experimental period was observed.

In the current study, we saw a significant increase in the expansion of CD8α+ T cell population in resiquimod pretreated lungs as compared with PBS pretreated control lungs. Although, the mechanisms underlying the ability of resiquimod to increase CD8α+ T cell recruitment in the absence of antigen stimulation is unclear, evidence of a different TLR7 agonist, promoting proliferation of naïve T cells has been observed in vitro in the presence of antigen presenting cells, dendritic cells (DCs) [26]. In addition to CD8α+ T cell recruitment, macrophage recruitment has also been higher in the lungs following pre-hatch delivery of resiquimod. Previously, avian macrophages have been shown to elicit an antiviral response in vitro against avian influenza virus replication [27].

Although it has been shown that type 1 IFNs are induced following resiquimod treatment in other animal models [28], our previous work indicated the lack of production of type 1 IFNs and increased production of IL-1β following treatment of avian macrophage with resiquimod [15,27]. In agreement with the observations of these later studies, a lack of production of type IFNs have been shown in avian macrophages following stimulation of these cells with resiquimod [29]. Although avian macrophages lack expression of type 1 IFNs following resiquimod treatment, macrophages are capable of expressing type 1 IFN mRNA expression in response to other TLR ligands [30,31] However, resiquimod has been shown to produce type 1 IFN mRNA in peripheral blood mononuclear cells (PBMC) originated from chickens [9,32] and this indicates that we require further investigations to observe type 1 IFN response in our in vivo model.

The significant upregulation of IFN-γ mRNA was seen in resiquimod pretreated lungs and suggests that resiquimod is capable of promoting the production of cytokines that facilitate Th1 biased adaptive immune response [33,34]. Since macrophages and CD8α+ T cells are known to be sources of IFN-γ [35,36], it is possible that the observed IFN-γ mRNA expression in our study could have come from the recruited T cells and macrophages. 

Another cytokine mRNA that was induced by resiquimod in our study that could have played a role in recruitment of immune cells in lungs is the IL-1β. It has been observed that through TLR stimulation, activated macrophages can secrete IL-1β among other chemokines and cytokines [30,37]. Furthermore, IL-1β is a known chemotactic cytokine that traffics macrophages and other immune cells into sites of an injury [38]. This autocrine positive feedback loop could possibly explain the increased recruitment of macrophages into the lung tissue along with an associated increase in IL-1β that we observed. IL-1β, originating from avian macrophages, has been shown to elicit antiviral response [27] and it is possible that IL-1β expression in lungs could have played a role in reducing IBV shedding early during the infection.

Correlating with the recruitment of immune cells and upregulation of selected cytokine mRNA by the time of IBV infection (day of hatch), we saw a significant reduction in IBV virus shedding through both oral and fecal routes early during the study in resiquimod pretreated chickens as compared with PBS pretreated chickens. Supporting our findings, in chickens, two previous studies have shown that prophylactic administration of resiquimod to two-week-old chickens, led to significant reduction in IBDV replication [10] and shedding of AIV [16]. Our previous work also recorded that in ovo administration of resiquimod is capable of reducing cloacal shedding of ILTV genome loads [15].

## 5. Conclusions

We have shown that pre-hatch delivery of resiquimod, increased IFN-γ and IL-1β gene upregulation, and CD8α+ T cell and macrophage recruitments into the lung tissue of chickens. Next, we found that when resiquimod was administered along with IBV killed vaccine in ovo, resiquimod could act as an adjuvant increasing cell-mediated immune response. The enhanced immune response by in ovo delivered resiquimod correlates with reduced IBV genome loads in cloacal and oropharyngeal routes following IBV encounter post-hatch.

## Supplementary Materials

The following is available online at https://www.mdpi.com/2076-393X/8/2/186/s1, Table S1: PCR primers used in RT-PCR assays.
