# Supplementary material for: In Ovo Delivered Toll-Like Receptor 7 Ligand, Resiquimod Enhances Host Responses against Infectious Bronchitis Corona Virus (IBV) Infection
Source: Vaccines (Basel). 2020 Apr 15;8(2):186. doi: 10.3390/vaccines8020186 (PMC7349678; doi:10.3390/vaccines8020186)
Supplement: Supplementary file 1 [file vaccines-08-00186-s001.pdf]

vaccines

MDPI

Article

In Ovo Delivered Toll-Like Receptor 7 Ligand, Resiquimod Enhances Host Responses against Infectious Bronchitis Corona Virus (IBV) Infection

Upasama De Silva Senapathi [1], Mohamed Aboelkhair [2], Kekungu Puro [3], Mariam Ali [1], Aruna Amarasinghe [1], M. Sarjoon Abdul-Cader [1], Guido Van Marle [4], Markus Czub [1] and Mohamed Faizal Abdul-Careem [1,*]

Table S1. PCR primers used in RT-PCR assays.

Primer	Sequence (5′-3′)	Fragment (bps)	Reference
IBV-N	F-GACGGAGGACCTGATGGTAA<br>R-CCCTTCTTCTGCTGATCCTG	206	[19]
β-actin	F-CAACACAGTGCTGTCTGGTGGTA<br>R-ATCGTACTCCTGCTTGCTGATCC	205	[19]
IL-1β	F-GTGAGGCTCAACATTGCGCTGTA<br>R- TGTCCAGGCGGTAGAAGATGAAG	214	[19]
iNOS	F-GGCAGCAGCGTCTCTATGACTTG<br>R-GACTTTAGGCTGCCCAGGTTG	185	[19]
IFN-γ	F-ACACTGACAAGTCAAAGCCGCACA<br>R-AGTCGTTCATCGGGACCTTGGC	129	[19]

© 2020 by the authors. Submitted for possible open access publication under the terms and conditions of the Creative Commons Attribution (CC BY) license (http://creativecommons.org/licenses/by/4.0/).
